# New records of *Ctenolepismacalvum* (Ritter,1910) (Zygentoma, Lepismatidae) from Japan

**DOI:** 10.3897/BDJ.10.e90799

**Published:** 2022-11-03

**Authors:** Megumi Shimada, Hiroki Watanabe, Yukio Komine, Rika Kigawa, Yoshinori Sato

**Affiliations:** 1 Tokyo National Research Institute for Cultural Properties, Tokyo, Japan Tokyo National Research Institute for Cultural Properties Tokyo Japan; 2 Kyushu National Museum, Fukuoka, Japan Kyushu National Museum Fukuoka Japan; 3 Nara National Museum, Nara, Japan Nara National Museum Nara Japan

**Keywords:** *Ctenolepismacalvum*, Japan, COI, EMBL/GenBank/DDBJ, Lepismatidae, biological invasion, household pests, museum pests

## Abstract

**Background:**

Silverfish are known as one of the major pests which feed on paper and starch-based materials and can cause serious problems in museums, libraries and archives.

*Ctenolepismacalvum* (Ritter, 1910) was first recorded from Ceylon (now Sri Lanka) and has also been known from Central American countries including Guyana and Cuba. Recently, its rapid spread to European countries, including Austria, Czech, Germany and Norway, has been reported. In addition, there are unverified records of *C.calvum* from 17 more countries in the on-line citizen-science platforms iNaturalist.

**New information:**

We report *C.calvum* in Japan for the first time, from Hokkaido, Miyagi, Tokyo, Fukuoka and Nagasaki Prefectures. The specimens in Japan were observed in detail by stereomicroscope, optical microscope and scanning electron microscope. The occurrence of this species is a serious problem from the viewpoint of protection of cultural properties. We also registered their mitochondrial cytochrome oxidase I (COI) gene in EMBL/GenBank/DDBJ.

## Introduction

Silverfish is one of the most well-known and important insect pests in museums, libraries and archives which hold ancient documents. They often eat the surface of paper embedded with starch pastes and damage traditional paintings and documents. In Japan, *Lepismasaccharinum* Linnaeus, 1758, *Thermobiadomestica* (Packard, 1873), *Acrotelsacollaris* (Fabricius, 1793) and three species of *Ctenolepisma — C.villosum* (Fabricius, 1775), *C.longicaudatum* Escherich, 1905 and *C.lineatum* (Fabricius, 1775) — are known (Machida and Masumoto 2006). Especially *L.saccharinum* and *C.villosum* are common species. These species usually have low reproductive ability at ambient and lower relative humidity (RH). Therefore, the damage caused has not been considered very significant in the buildings with air conditioning systems. However, a species of silverfish which can reproduce quickly under ambient RH conditions was discovered in several Japanese museums and libraries. Here, we identified and reported this silverfish and registered their COI gene in EMBL/GenBank/DDBJ.

## Materials and methods

### Specimens examined

Silverfish samples were collected from several museums and libraries. Some of them were collected by hand picking or insect aspirator and others were trapped by blunder traps. The silverfish were captured in areas where temperature and humidity were controlled around 20-30°C and 50-60% RH, respectively. Trapped specimens were removed from traps with hexane and stored in 99% ethanol. They were used for molecular examination. Alive individuals were bred in humidity-controlled cages. Some of them and their descendants were treated and stored in 80% ethanol and used for both morphological and molecular examinations.

### Sampling, DNA extraction, PCR and DNA sequencing

DNA was extracted from some legs, antennae or caudal appendages separated from the body using a Qiagen DNeasy Blood and Tissue Kit (Qiagen, Inc., Valencia, California, USA). The specimens used for DNA extraction were deposited in the collection of Tokyo National Research Institute for Cultural Properties (TBK), Tokyo, Japan. Polymerase chain reaction (PCR) amplification of COI was carried out using a TaKaRa PCR Thermal Cycler Dice (Takara Bio Inc., Kusatsu, Shiga, Japan) with an amplification programmed: 3 minutes at 95°C, 35 cycles of 20 seconds at 98°C, 15 seconds at 55°C, 1 minute at 72°C and a final extension step for 5 minutes at 72°C. A single fragment of 658 bp was amplified from the PCR amplification using the primer pair LCO1490 (5’-GGTCAACAAATCATAAAGATATTGG-3’) and HCO2198 (5’-TAAACTTCAGGGTGACCAAAAAATCA-3’) (Folmer et al. 1994). KAPA HiFi HotStart ReadyMix PCR Kit (Nippon Genetics Co., Ltd., Tokyo, Japan) was used for the enzyme. PCR products were visualised via 2% agarose gel electrophoresis with Midori Green Direct (Nippon Genetics Co., Ltd., Tokyo, Japan) to confirm their amplification. PCR products were purified with NucleoSpin Gel and PCR Clean Up Kit (Takara Bio Inc., Kusatsu, Shiga, Japan) according to the manufacturer’s instructions. Purified PCR products were sequenced by Macrogen Japan Sequencing Service (Macrogen Japan Corp.; https://dna.macrogen.com/eng/index.jsp). The DNA sequence was edited with Chromas Version 2.6.6 (Technelysium Pty. Ltd.; https://technelysium.com.au) and MEGA (Molecular Evolutionary Genetics Analysis) Version 11 (Tamura et al. 2021).

### Morphological observation

Morphological data were mainly taken from specimens stored in 80% ethanol and slide-mounted specimens, using the Leica MZ125 high-performance stereomicroscope (Leica Microsystems GmbH, Wetzlar, Germany) and Olympus BX53 (Olympus Corp., Tokyo, Japan). Specimens stored in 80% ethanol were treated in 20% potassium hydroxide (KOH) for 5-6 hours at room temperature, substances in the body were removed with glacial acetic acid/Clear Lite 3/absolute ethanol (2:2:1) and the specimens were dehydrated with a graded alcohol series before being slide-mounted in Euparal. Some specimens were stained with acid fuchsin. Photographs were taken with a Leica DFC290 HD (Leica Microsystems GmbH, Wetzlar, Germany) and Nikon D5300 (Nikon Corp., Tokyo, Japan) camera. Image J version 1.53c (U.S. National Institutes of Health, Bethesda, Maryland, U.S.A.) was used to take measurements. The final images were prepared with Photoshop CC2021 and Illustrator CC2021 (Adobe Inc., San Jose, California, U.S.A.).

### Scanning electron microscope

Nine specimens (8 adults and 1 juvenile) of *C.calvum* were used for scanning electron microscope (SEM) examination. Specimens examined were stored in 80% ethanol and dehydrated with a graded alcohol series, then the specimens were air dried prior to mounting on 10 mm aluminium stubs. Specimens were held in place with carbon double-sided tape (Nisshin EM Co., Ltd., Tokyo, Japan) and coated with gold in a Quick Auto Coater SC-701AT (Sanyu Denshi Co., Ltd., Tokyo, Japan) for 60 seconds. We used an S-3700N scanning electron microscope (Hitachi Ltd., Tokyo, Japan) and photographed screen images.

### Nucleotide sequence accession number

The COI gene sequences of *Ctenolepismacalvum* 22TBK001-22TBK004 have been deposited in the EMBL/GenBank/DDBJ databases under the accession number LC719153-LC719156.

## Taxon treatments

### Ctenolepisma calvum

Ritter, 1910

14055CAA-5A80-565E-A9C7-CEE435F26399

#### Materials

- **Type status:** Other material. **Occurrence:** individualCount: 1; sex: female; lifeStage: adult; preparations: DNA extraction; occurrenceID: 3D24CB02-5821-50A9-B4F5-754AEC474BF8; **Taxon:** scientificName: *Ctenolepismacalvum*; kingdom: Animalia; phylum: Euarthropoda; class: Insecta; order: Zygentoma; family: Lepismatidae; genus: Ctenolepisma; specificEpithet: *calvum*; scientificNameAuthorship: Ritter; **Location:** country: Japan; countryCode: JP; stateProvince: Hokkaido; **Identification:** identifiedBy: Megumi Shimada; dateIdentified: 2022; **Event:** samplingProtocol: blunder trap; samplingEffort: 1 month; startDayOfYear: 2021-11; endDayOfYear: 2021-12; **Record Level:** institutionCode: TBK
- **Type status:** Other material. **Occurrence:** recordedBy: Aya Moriya; individualCount: 9; sex: female; lifeStage: adults and juveniles; occurrenceID: 325672A5-B4E0-5FD9-8416-04846F75664D; **Taxon:** scientificName: *Ctenolepismacalvum*; kingdom: Animalia; phylum: Euarthropoda; class: Insecta; order: Zygentoma; family: Lepismatidae; genus: Ctenolepisma; specificEpithet: *calvum*; scientificNameAuthorship: Ritter; **Location:** country: Japan; countryCode: JP; stateProvince: Miyagi Prefecture; municipality: Tagajo-shi; **Identification:** identifiedBy: Megumi Shimada; dateIdentified: 2022; **Event:** samplingProtocol: blunder trap; samplingEffort: 1 month; startDayOfYear: 2021-10-22; endDayOfYear: 2021-11-22; **Record Level:** institutionCode: TBK
- **Type status:** Other material. **Occurrence:** recordedBy: Megumi Shimada and Yoshinori Sato; individualCount: 4; sex: female; lifeStage: adults and juveniles; occurrenceID: D49EDB94-7982-57C8-92F5-E288AD8D178D; **Taxon:** scientificName: *Ctenolepismacalvum*; kingdom: Animalia; phylum: Euarthropoda; class: Insecta; order: Zygentoma; family: Lepismatidae; genus: Ctenolepisma; specificEpithet: *calvum*; scientificNameAuthorship: Ritter; **Location:** country: Japan; countryCode: JP; stateProvince: Tokyo; municipality: Taito-ku; **Identification:** identifiedBy: Megumi Shimada; dateIdentified: 2021; **Event:** samplingProtocol: hand picking; eventDate: 2021-4-14
- **Type status:** Other material. **Occurrence:** individualCount: 25; sex: female; lifeStage: adults and juveniles; occurrenceID: B6E97034-B389-5279-997C-9D4AE36B2394; **Taxon:** scientificName: *Ctenolepismacalvum*; kingdom: Animalia; phylum: Euarthropoda; class: Insecta; order: Zygentoma; family: Lepismatidae; genus: Ctenolepisma; specificEpithet: *calvum*; scientificNameAuthorship: Ritter; **Location:** country: Japan; countryCode: JP; stateProvince: Tokyo; municipality: Taito-ku; **Identification:** identifiedBy: Megumi Shimada; dateIdentified: 2021; **Event:** samplingProtocol: insect aspirator; startDayOfYear: 2021-4; endDayOfYear: 2021-10
- **Type status:** Other material. **Occurrence:** individualCount: 28; sex: female; lifeStage: Adults and juveniles; occurrenceID: 9CBA9194-AD9C-5CC4-9F6C-CD797FA73061; **Taxon:** scientificName: *Ctenolepismacalvum*; kingdom: Animalia; phylum: Euarthropoda; class: Insecta; order: Zygentoma; family: Lepismatidae; genus: Ctenolepisma; specificEpithet: *calvum*; scientificNameAuthorship: Ritter; **Location:** country: Japan; countryCode: JP; stateProvince: Tokyo; municipality: Taito-ku; **Identification:** identifiedBy: Megumi Shimada; dateIdentified: 2021; **Event:** samplingProtocol: blunder trap; startDayOfYear: 2021-5; endDayOfYear: 2021-7
- **Type status:** Other material. **Occurrence:** recordedBy: Toshiyuki Torigoe; individualCount: 5; sex: female; lifeStage: Adults and juveniles; occurrenceID: CA9F5623-9FCE-57B2-8C34-7246CB6E074B; **Taxon:** scientificName: *Ctenolepismacalvum*; kingdom: Animalia; phylum: Euarthropoda; class: Insecta; order: Zygentoma; family: Lepismatidae; genus: Ctenolepisma; specificEpithet: *calvum*; scientificNameAuthorship: Ritter; **Location:** country: Japan; countryCode: JP; stateProvince: Tokyo; municipality: Taito-ku; **Identification:** identifiedBy: Megumi Shimada; dateIdentified: 2021; **Event:** samplingProtocol: blunder trap; samplingEffort: 3 months; startDayOfYear: 2021-4-14; endDayOfYear: 2021-7-6; **Record Level:** institutionCode: TBK
- **Type status:** Other material. **Occurrence:** recordedBy: Ikari Shodoku Co., Ltd., Tokyo, Japan; individualCount: 2; sex: female; lifeStage: juveniles; preparations: DNA extraction; occurrenceID: F527E65B-6535-57C8-A83B-C00156D0D6C6; **Taxon:** scientificName: *Ctenolepismacalvum*; kingdom: Animalia; phylum: Euarthropoda; class: Insecta; order: Zygentoma; family: Lepismatidae; genus: Ctenolepisma; specificEpithet: *calvum*; scientificNameAuthorship: Ritter; **Location:** country: Japan; countryCode: JP; stateProvince: Tokyo; municipality: Taito-ku; **Identification:** identifiedBy: Megumi Shimada; dateIdentified: 2021; **Event:** samplingProtocol: hand picking; eventDate: 2021-7-8; **Record Level:** institutionCode: TBK
- **Type status:** Other material. **Occurrence:** recordedBy: Ikari Shodoku Co., Ltd., Tokyo, Japan; individualCount: 4; sex: female; lifeStage: juveniles; preparations: DNA extraction; occurrenceID: 989F9F50-0AB8-5882-B223-DACB9EE034F9; **Taxon:** scientificName: *Ctenolepismacalvum*; kingdom: Animalia; phylum: Euarthropoda; class: Insecta; order: Zygentoma; family: Lepismatidae; genus: Ctenolepisma; specificEpithet: *calvum*; scientificNameAuthorship: Ritter; **Location:** country: Japan; countryCode: JP; stateProvince: Tokyo; municipality: Taito-ku; **Identification:** identifiedBy: Megumi Shimada; dateIdentified: 2022; **Event:** samplingProtocol: blunder trap; samplingEffort: 2 months; startDayOfYear: 2021-10-27; endDayOfYear: 2021-12-27; **Record Level:** institutionCode: TBK
- **Type status:** Other material. **Occurrence:** recordedBy: Hiroki Watanabe; individualCount: 19; sex: female; lifeStage: Adults and juveniles; occurrenceID: 94AF841C-2725-5BE9-BCB6-BB2C5E86A315; **Taxon:** scientificName: *Ctenolepismacalvum*; kingdom: Animalia; phylum: Euarthropoda; class: Insecta; order: Zygentoma; family: Lepismatidae; genus: Ctenolepisma; specificEpithet: *calvum*; scientificNameAuthorship: Ritter; **Location:** country: Japan; countryCode: JP; stateProvince: Fukuoka Prefecture; municipality: Dazaifu-shi; **Identification:** identifiedBy: Megumi Shimada; dateIdentified: 2021; **Event:** samplingProtocol: hand picking; startDayOfYear: 2019-7-4; endDayOfYear: 2019-10-30
- **Type status:** Other material. **Occurrence:** recordedBy: Atsushi Morizono; individualCount: 1; sex: female; lifeStage: juvenile; preparations: DNA extraction; occurrenceID: 7EB1A43B-75C1-5718-B105-B7C43AC5BCF4; **Taxon:** scientificName: *Ctenolepismacalvum*; kingdom: Animalia; phylum: Euarthropoda; class: Insecta; order: Zygentoma; family: Lepismatidae; genus: Ctenolepisma; specificEpithet: *calvum*; scientificNameAuthorship: Ritter; **Location:** country: Japan; countryCode: JP; stateProvince: Nagasaki Prefecture; municipality: Nagasaki-shi; **Identification:** identifiedBy: Megumi Shimada; dateIdentified: 2022; **Event:** samplingProtocol: blunder trap; samplingEffort: 1 month; startDayOfYear: 2021-10-31; endDayOfYear: 2021-11-30; **Record Level:** institutionCode: TBK

#### Diagnosis

**Figure 1.**
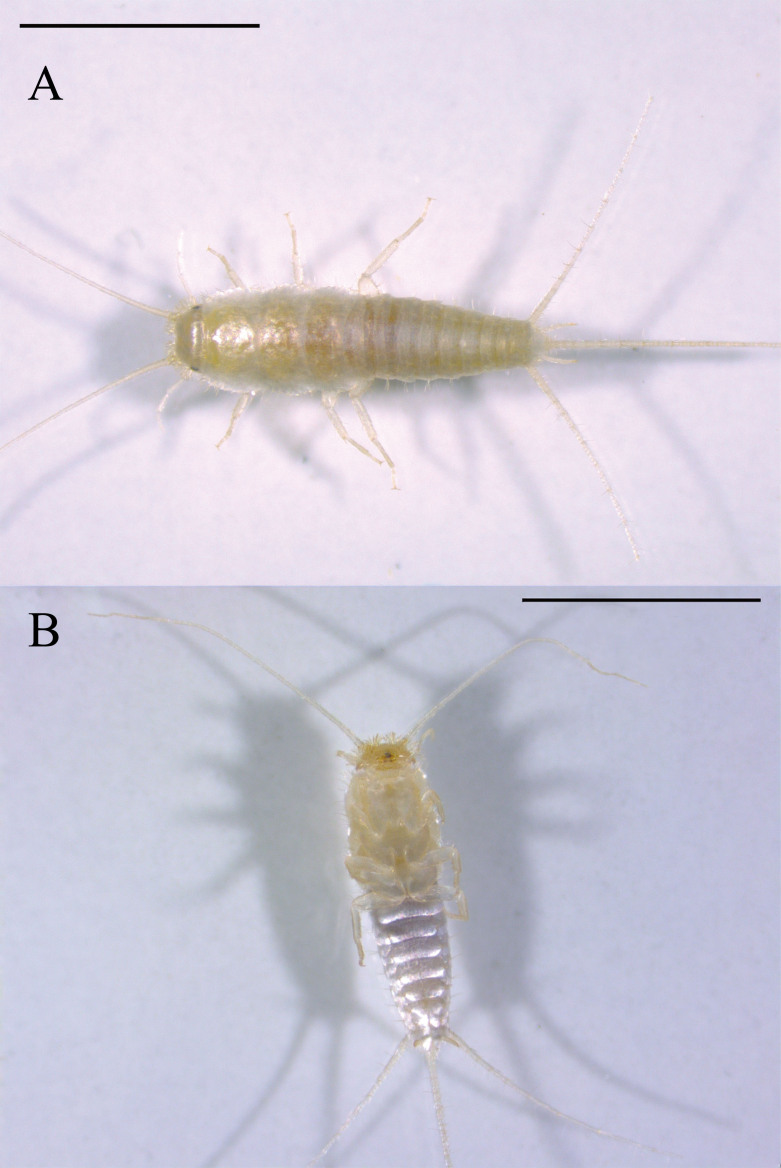
*Ctenolepismacalvum* (Taito-ku, Tokyo): **A** dorsal view; **B** ventral view. Scale bar = 5 mm.

**Figure 2.**
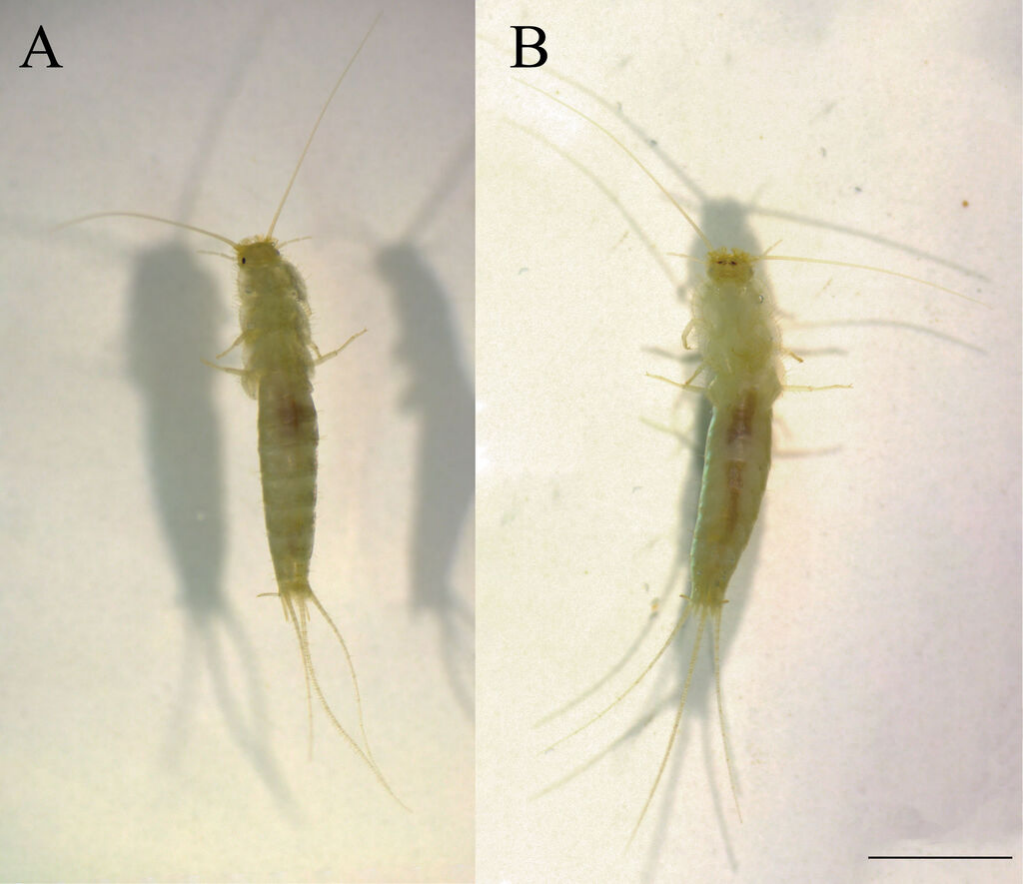
*Ctenolepismacalvum* (Dazaifu-shi, Fukuoka Prefecture) in alcohol: **A** dorsal view; **B** ventral view. Scale bar = 5 mm.

**Figure 3.**
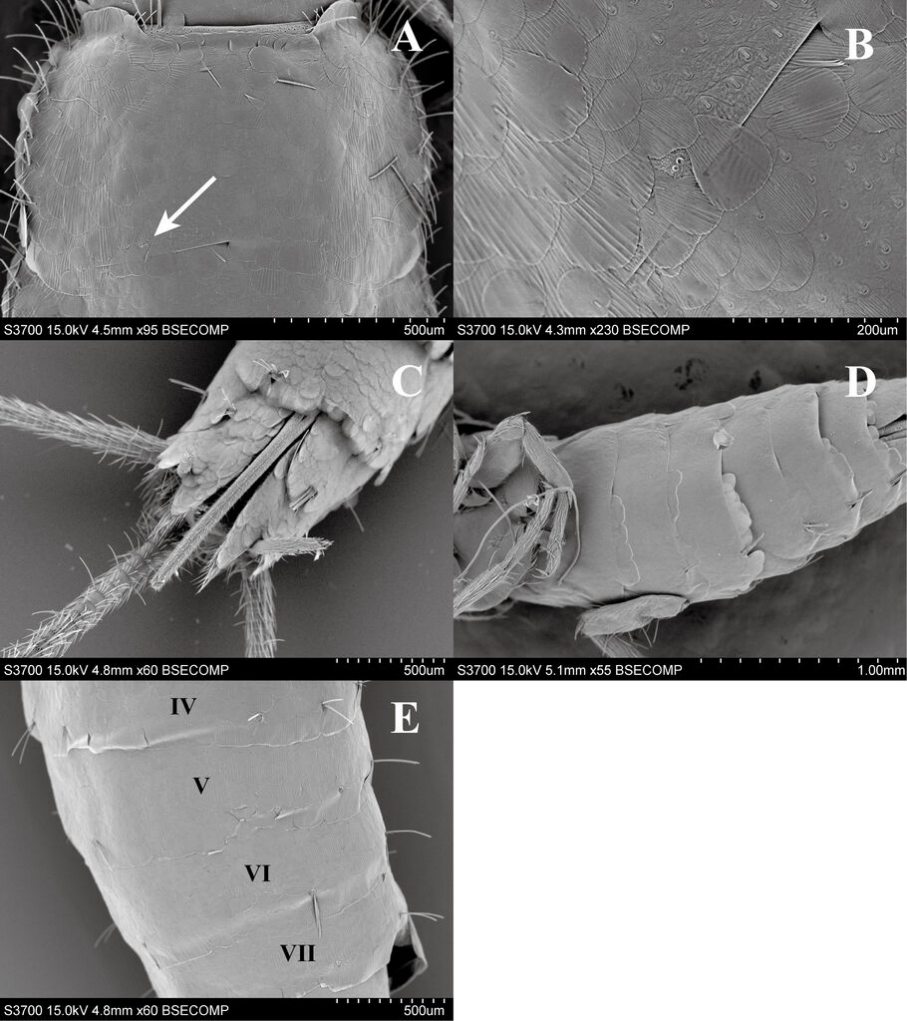
*Ctenolepismacalvum* (Dazaifu-shi, Fukuoka Prefecture), scanning electron microscopy micrographs: **A** dorsal view of pronotum; **B** macrochaetae root of prothorax; **C** ventral rear of abdomen; **D** ventral view of abdomen; **E** dorsal view of abdomen.

*Ctenolepismacalvum* can be distinguished from other species of *Ctenolepisma* in Japan by a combination of the following characters: whitish colour (Fig. 1), but yellowish-brown colour in alcohol and a little translucent (Fig. 2); posterior margin of the thoracic nota with 2 macrochaetae on each side (Fig. 3A and B), but immature individuals have 1 macrochaeta; only one pair of abdominal styli (Fig. 3C); absence of median urosternal combs of macrochaetae (Fig. 3D); 3+3 bristle-combs in the abdominal tergites II–V, 2+2 bristle-combs in the abdominal tergites VI–VIII (Fig. 3E); last abdominal tergite trapezoidal and shorter than wide (Fig. 3C); short ovipositor (Fig. 3C).

The lateral caudal appendages approximately two-thirds of body length and median caudal appendage approximately as long as body.

#### Distribution

Ceylon, now Sri Lanka (Ritter 1910, Crusz 1957), Guyana, Cuba (Wygodzinsky 1972), Austria, Czech, Germany, Norway (Landsberger and Querner 2018, Hage et al. 2020, Kurz 2020, Kulma et al. 2022) and Japan (present study). There are additional records of *C.calvum* from Croatia, Finland, Italy, Kosovo, Luxembourg, Mexico, Portugal, Russia, Singapore, Slovakia, Slovenia, Spain, Switzerland, Turkey, Ukraine, Venezuela and Vietnam, based on photographic identification in the on-line citizen-science platforms iNaturalist accessed on 30 Sep 2022 (https://www.inaturalist.org/), although these data may not be reliable because detailed examinations on diagnostic characters using optical microscope or scanning electron microscope were not conducted.

#### Remarks

After the first description of *C.calvum* by Ritter (1910), only Kulma et al. (2022) provided additional morphological data on the species reported from Europe. The material from Japan studied by us is identical with the morphology of *C.calvum* in Europe.

As in Ritter (1910) and Kulma et al. (2022), individuals of *C.calvum* found in museums and libraries in Japan were up to approximately 8 mm, but breeding populations were up to 12 mm. Permission to publish detailed data, such as the name and location of the institutions such as museums and libraries, was not obtained from them.

## Discussion

**Figure 4.**
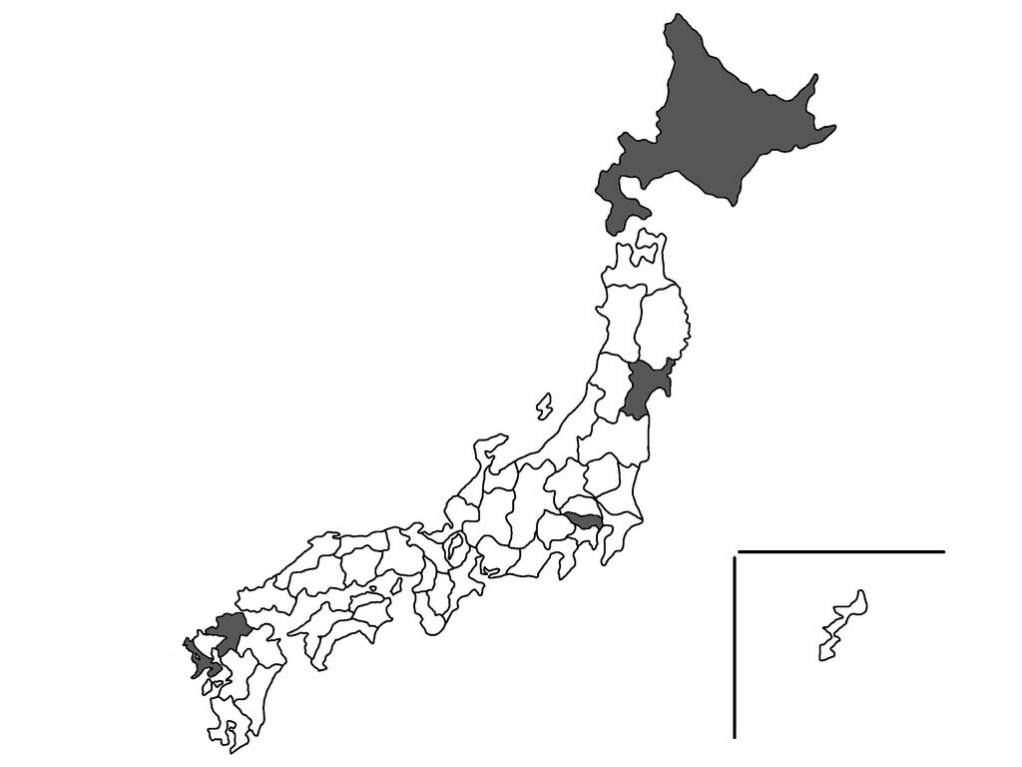
The areas where *Ctenolepismacalvum* was discovered as of May 2022. The Prefectures in which *C.calvum* was found are coloured.

No male *C.calvum* was observed so far amongst the captured individuals and bred populations in our laboratories in Japan. It is possible that the male sex ratio is extremely low and/or that they can reproduce parthenogenetically (Watanabe et al., unpublished data). If *C.calvum* can reproduce parthenogenetically, they should have strong fecundity. Rapid proliferation of this species seen in several cases is a characteristic different from that seen with other species of silverfish in Japan, such as *C.villosum*, *C.longicaudatum* and *L.saccharinum*. In recent years, *C.calvum* has been spreading rapidly in Europe (Kulma et al. 2022 and Querner et al. 2022). Besides, according to iNaturalist, there are 60 reports of *C.calvum* from 16 countries in Europe from 2018. In Japan, since areas where *C.calvum* was found were distributed from North to South (Fig. 4), the species may have already prevailed in vast areas in Japan. As *C.calvum* was not included in the list of known silverfish species in Japan described by Machida and Masumoto (2006), they may have invaded recently and spread rapidly in Japan. Further research on distribution and genetic differentiation of *C.calvum* in Japan is necessary to identify when and how they invaded.

In this study, we sequenced a part of the COI gene (658bp) collected from bred populations, which was registered at EMBL/GenBank/DDBJ. Genetic information on family Lepismatidae has not been registered in a large number yet. In the Lepismatidae data of NCBI, individuals with much genetic variations, which would normally be considered as of different species, are grouped as the same species. It is possible that morphological misidentification caused this situation. In order to ensure gene registration to EMBL/ GenBank/DDBJ, based on precise identification, further research on Lepismatidae will be necessary.

## Supplementary Material

XML Treatment for Ctenolepisma calvum
